# Metabolomic Analysis of Extracellular Vesicles from the Cereal Fungal Pathogen *Fusarium graminearum*

**DOI:** 10.3390/jof9050507

**Published:** 2023-04-24

**Authors:** Donovan Garcia-Ceron, Thy T. Truong, Julian Ratcliffe, James A. McKenna, Mark R. Bleackley, Marilyn A. Anderson

**Affiliations:** 1 Department of Biochemistry and Chemistry, La Trobe Institute for Molecular Science, La Trobe University, Bundoora 3086, Australia; d.garcia-ceron@latrobe.edu.au (D.G.-C.);; 2 Proteomics and Metabolomics Platform, School of Agriculture, Biomedicine, and Environment, La Trobe University, Bundoora 3086, Australia; 3 La Trobe Bioimaging Platform, La Trobe Institute for Molecular Science, La Trobe University, Bundoora 3086, Australia

**Keywords:** extracellular vesicles, fungal EVs, fungi, yeast, *Fusarium graminearum*, metabolomics, zearalenone, mycotoxins, unconventional secretion, fungal pathogen, plant pathogen

## Abstract

*Fusarium graminearum* (*F. graminearum*) is a filamentous fungus that infects cereals such as corn, wheat, and barley, with serious impact on yield as well as quality when the grain is contaminated with mycotoxins. Despite the huge impact of *F. graminearum* on food security and mammalian health, the mechanisms used by *F. graminearum* to export virulence factors during infection are not fully understood and may involve non-classical secretory pathways. Extracellular vesicles (EVs) are lipid-bound compartments produced by cells of all kingdoms that transport several classes of macromolecules and are implicated in cell–cell communication. EVs produced by human fungal pathogens carry cargo that facilitate infection, leading us to ask whether plant fungal pathogens also deliver molecules that increase virulence via EVs. We examined the metabolome of the EVs produced by *F. graminearum* to determine whether they carry small molecules that could modulate plant–pathogen interactions. We discovered that EVs from *F. graminearum* were produced in liquid medium-containing inducers of trichothecene production, but in lower quantities compared to other media. Nanoparticle tracking analysis and cryo-electron microscopy revealed that the EVs were morphologically similar to EVs from other organisms; hence, the EVs were metabolically profiled using LC-ESI-MS/MS. This analysis revealed that EVs carry 2,4-dihydroxybenzophenone (BP-1) and metabolites that have been suggested by others to have a role in host–pathogen interactions. BP-1 reduced the growth of *F. graminearum* in an in vitro assay, suggesting that *F. graminearum* might use EVs to limit metabolite self-toxicity.

## 1. Introduction

The filamentous fungus *Fusarium graminearum* (*F. graminearum*) is a serious pathogen of wheat, corn, barley, and soybean [1,2]. These crops are crucial to global food security; hence, *F. graminearum* has been recognized as the fourth most important plant pathogen [3]. Control of *F. graminearum* is restricted by the lack of effective disease-resistant cultivars and is largely dependent on the use of chemical fungicides [4]. However, concerns about the increase in resistance of fungal pathogens to chemical fungicides as well as environmental and health risks [5] have led to an urgent need to develop the next generation of antifungals for *F. graminearum* management. The negative impact of *F. graminearum* on yield is compounded by contamination of the grain by mycotoxins, secondary metabolites that are toxic to mammals [6].

Although the contamination of grain by *F. graminearum* is a global problem that affects food security, the mechanisms for export of toxic metabolites are not fully understood [7]. Extracellular vesicles (EVs) offer one potential pathway for the export of these metabolites. Recently, the production of EVs has been linked to the transport of pigments, protein effectors, peptide mycotoxins, and other molecules important for fungal infections [8,9,10,11,12]. Fungal EVs are between 30 and 1000 nm in size and often transport macromolecules that function in cell communication [13,14]. This is well-described for yeast pathogens that infect humans [11,15], although the function of EVs in plant pathogenic fungi is relatively unexplored. In an earlier study we discovered that a tailored growth medium with amino acids and not sugars as a carbon source was essential for the isolation of EVs from *F. graminearum*. These EVs contained protein effectors that are expressed during infection of corn, including potential effectors that lack a signal peptide [10]. The observation that EVs from *F. oxysporum* cause a hypersensitive response in cotton cotyledons and are likely to transport phytotoxic pigments [8] led to the question of whether EVs are also a mechanism for the unconventional secretion of metabolites that may enhance virulence in *F. graminearum*.

In this study we performed an untargeted metabolomic analysis on the EVs produced by *F. graminearum*, as well as in the soluble-secreted fraction (secretome) and the whole-cell lysate, to determine which metabolites were enriched in EVs. The EV metabolome contained molecules with previously described antibacterial and antifungal roles; thus, we performed antimicrobial assays using bacteria and fungi to explore whether components of the EV metabolome work in cell competition. LC-ESI-MS/MS did not detect trichothecenes or zearalenone in the EV preparations. Instead, zearalenone was detected by ELISA in EVs and not in EV depleted samples, suggesting that EVs may be involved in the transport of hydrophobic compounds involved in host–pathogen interactions.

## 2. Materials and Methods

### 2.1. Microbial Cultures

*Fusarium graminearum* (*F. graminearum*) strain PH-1 was a gift from Dr. Kim Hammond-Kosack (Rothamsted Research, Harpenden, Herts, UK). *F. graminearum* was grown in liquid medium using yeast nitrogen base (YNB, no carbohydrates, no amino acids, with ammonium sulphate), supplemented with L-arginine (0.5 g/L, Sigma, St. Louis, MO, USA) and putrescine dihydrochloride (5 mM, Sigma). The components were dissolved in ultrapure water (final pH 3.5), filtered through a 0.22 µm Steritop (Merck, Kenilworth, NJ, USA), and stored at 4 °C until use. This medium was named “YNB+putrescine”. Extracellular vesicles (EVs) were recovered from 500 mL of liquid culture that had been inoculated with 10^4^ spores/mL in a 2 L baffled flask. The cultures were incubated for 5 days at 25 °C with agitation of 100 rpm. After incubation, the mycelia were removed by filtration through Miracloth (Merck) and discarded, and the supernatant was filtered again to remove insoluble material using 0.45 µm regenerated cellulose membranes (HAWP, Merck), followed by concentration to around 500 µL using centrifugal filter units with a size cut-off (MWCO) of 100 kDa (Merck). The eluate from this step was discarded except for a sample taken for zearalenone detection by ELISA.

*Staphylococcus aureus* (ATCC 9144) and *Escherichia coli* (BW25113) cells were maintained on solid Luria-Bertani (LB) medium. A single colony was inoculated into a 3 mL shaking culture of LB broth and incubated overnight at 37 °C at 300 rpm. *Candida albicans* (ATCC 90028) was maintained on solid yeast peptone dextrose (YPD) and a single colony was inoculated into a 3 mL shaking culture of YPD and incubated overnight at 30 °C at 300 rpm.

### 2.2. Isolation of Extracellular Vesicles (EVs)

EVs were recovered by size-exclusion chromatography as described by [10]. Briefly, the 500 µL of concentrated supernatant was mixed with the lipophilic dye FM5-95 (1.75 µM, Thermo Fisher, Waltham, MA, USA) and incubated for 15 min at room temperature protected from light. Simultaneously, a 20 mL plastic column (Takara, Kusatsu, Shiga, Japan) was loaded with 10 mL of Sepharose CL 2B (Sigma) and equilibrated with Dulbecco’s phosphate-buffered saline (DPBS, Billings, MT, USA). The supernatant was placed onto the Sepharose, and 45 fractions (approx. 300 µL each) were eluted in DPBS and collected in a black microtiter plate with black bottom (Greiner Bio-One, Kremsmünster, Austria). The fluorescence of the fractions was measured immediately in a SpectraMax M2 plate reader (Molecular Devices, San Jose, CA, USA). Adjacent fractions with consistent positive relative fluorescence units (RFUs) above 1.5 were pooled and labeled “EV sample”. The protein concentration of the EV sample was measured with a Qubit4 (Thermo Fisher). The protein content of the unpooled fractions was quantified by microBCA (Thermo Fisher). All samples were snap-frozen and preserved at −80 °C until further use.

### 2.3. Preparation of Secretome (SEC) and Whole-Cell Lysate (WCL)

These samples were obtained as reported previously [16]. The SEC was prepared by concentrating 50 mL of the 0.45 µm filtered culture supernatant to 1 mL using 3 kDa MWCO centrifugal units (Merck). The WCL was prepared by grinding 80 mg of mycelia in 1 mL of DPBS with 70 mg of glass beads (710–1180 µm, Sigma) on a TissueLyser (Qiagen, Hilden, Germany), grinding for 30 s intervals at a frequency of 30/s, three total times, incubating in an ice bath every 30 s. The ground material was centrifuged at 21,130× *g* for 5 min at 4 °C and the supernatant was reserved for further analyses. The protein concentration of the WCL and SEC was determined immediately after collection with a Qubit4. Samples were snap-frozen and stored at −80 °C.

### 2.4. Detection of Zearalenone (ZEN) in EV Samples by ELISA

ZEN in EVs was determined using a Celer^®^ ZEA ELISA kit (Eurofins Technologies, Luxemburg) with minor modifications as suggested by the manufacturer. Briefly, EV samples were adjusted to 15 µg of protein in 100 µL using a Qubit4, before lysis with SDS (Sigma) at a final concentration of 0.5%. The lysed EVs were then mixed with 70% methanol in a final volume of 1 mL. Over a 5 min period, samples were alternated every 30 s between sonication at 4 °C and heating at 95 °C. The samples were then centrifuged at 21,130× *g* for 5 min at 4 °C, and the top 500 µL was carefully removed and retained for ELISA, following the manufacturer’s specifications. ZEN concentration was determined by absorbance at 450 nm versus the kit’s calibration curve in a SpectraMax M2 plate reader (Molecular Devices). The result for each sample was divided by a factor of five to correct the concentration applied to the standards by the manufacturer for 5× diluted cereal analysis. The ZEN concentration was obtained using a standard curve and an algorithm provided by Eurofins Technologies. The samples analyzed included EVs, the SEC fraction (50 mL of culture supernatant that was 3 kDa concentrated and 0.45 µm filtered), the WCL, 100 kDa eluate from the culture supernatant concentration step (see Section 2.1), EV-depleted samples (centrifuged at 100,000× *g* for 1 h), and PBS. Two technical replicates were included in each assay for EVs, EV-depleted, SEC, and WCL, and these were analyzed in triplicate.

### 2.5. Cryo-Electron Microscopy

A total of 300 mesh copper grids, coated with lacey formvar and carbon (ProSciTech, Townsville, Australia), were glow-discharged and transferred to an FEI Vitrobot (Thermo Fisher), followed by plunging into liquid ethane. A total of 5 µL of EVs at a protein concentration of 1 µg/µL (determined with Qubit4) were placed on the grid, and then the excess was blotted with filter paper. Grids were then plunged into ethane before being inserted into a Jeol JEM-2100 microscope running at 200 kV. Images were acquired with a Gatan Orius 200D camera running Digital Micrograph, version 2.32.888.0.

### 2.6. Sample Processing for Untargeted Metabolomic Analysis of Extracellular Vesicles (EVs)

Four biological replicates of EVs, secretome (SEC), and whole-cell lysate (WCL) were individually adjusted to 700 µL using DPBS and mixed with 800 µL of 80% methanol in water containing 8.7 mg/L of deuterated glucose (D-glucose-6,6-d2, Merck) as the internal standard. The samples were heated to 90 °C for 2 min for metabolite extraction, then cooled at room temperature for 45 min and centrifuged at 21,130× *g* for 5 min, retaining the supernatant. Each supernatant (100 µL) was 0.22 µm filtered and the filtrate was transferred to LC-ESI-MS/MS glass amber vials. To account for any cross-contamination during the extraction step, four quality control (QC) laboratory blanks were prepared with DPBS instead of sample and underwent the same extraction procedure as all samples. Five QC pooled samples (20 µL of all samples combined in the same vial), as well as a 12.5 mg/L QC standard mix (68 compounds) composed of sugar alcohols, organic acids, carbohydrates, and amino acids, along with QC methanol blanks, were analyzed to account for any biological degradation, carry-over contamination, and systemic fluctuations during the LC-ESI-MS/MS batch sequence analysis. Samples, QC pooled samples, QC laboratory blanks, and QC standards (7 μL) were injected onto an Agilent Zorbax Eclipse 1.8 μm XDB-C18 2.1 × 50 mm column. The column temperature was held constant at 40 ± 0.5 °C. Solvent A consisted of 0.1% aqueous formic acid and solvent B was composed of 80% methanol in water containing 0.1% formic acid. The untargeted polar metabolites were eluted with a linear gradient starting at 5% solvent B and held for 2 min, before steadily increasing to 95% solvent B over 16 min and held at 95% for 3 min before re-equilibrating to the starting solvent composition at 5% B for 8.5 min. The flow rate was 200 μL/min. The eluted metabolites from the column were introduced into the mass spectrometer via a heated electrospray ionization (HESI-II) probe and analyzed using an Orbitrap ID-X mass analyzer (Thermo Scientific, Waltham, MA, USA). The HESI was operated in the positive and negative ion polarities with parameters as follows: the electrospray voltage was 3.5 and 2.5 kV for positive and negative, respectively, and the ion transfer tube temperature was 300 °C. The vaporize temperature and the S-lens RF level were 275 °C and 30%, respectively. Ultra-high purity nitrogen was used as the sheath gas, auxiliary gas, and sweep gas at flows of 35 L/min, 7 L/min, and 0 L/min, respectively. Positive and negative ion polarity tandem mass spectrometry was carried out using the untargeted full scan MS and Data-Dependent MS2 (DDMS2) acquisition mode with the IQ-S Orbitrap mass resolution set at 120,000 and 15,000, respectively. The scan range was *m/z* 100–1000 at 1.0 micro-scan. The cycle time between master scans was 1.5 s. Stepped high collision-induced dissociation (HCD) energies were applied at 30, 45, and 55%. The automatic gain control (AGC) target value was set at 5.0 × 10^4^ counts, maximum accumulation time was 50 ms, and the isolation window was set at *m/z* 1.5. Data were acquired using the Thermo Scientific Xcalibur 4.4 software package.

### 2.7. Analysis of Untargeted Metabolomics Data

MS-DIAL (ver.4.8 database), specifically curated for untargeted metabolomic datasets, was used for peak detection, peak alignment, full scan MS and MS/MS spectral matching, and metabolite identification in accordance with the reporting standards set by metabolomics standard initiatives (MSI) [17,18]. Online mass spectral databases included MassBank, KEGG, Human Metabolome Database, METLIN, and PubChem. The measured exact mass, isotope pattern, and nitrogen rule were used to calculate the elemental compositions of the metabolites in MS Dial and were further validated using the mass assignment error (in ppm) equation. Therefore, features with exact masses outside of the −5.0 to 5.0 ppm mass error tolerance range were removed. Blank subtractions were carried out where peaks (features) detected in the laboratory blanks and methanol blanks were subtracted from the EV samples to remove false positives. Then, based on peak response and detection rate, the cut-off percentage relative standard deviation (RSD) for features detected in the QC pooled samples (*n* = 5) was set to a cutoff of ≤20%; hence, features with an RSD > 20% were removed. Metabolite concentrations (ng/mL) were calculated relative to arginine or xylitol standards (12.5 mg/L) for positive and negative modes, respectively, due to poor ionization of the deuterated glucose standard. Predicted metabolite solubility was calculated using ALOGPS 2.1 [19].

### 2.8. Statistical Analysis

The analysis was performed with MetaboAnalyst 5.0 [20], using the calculated metabolite relative concentration data (ng/mL) for “one factor” analysis. Missing values were imputated using the k-nearest neighbor (KNN) algorithm, with the feature-wise option. For multi-sample comparisons, samples were normalized using the QC pooled samples described in the sample preparation method following the probabilistic quotient normalization (PQR) [21]. Venn diagrams were created with Venny [22]. Samples were not normalized for pair-wise comparisons. All data were log_10_-transformed and scaled using the Pareto method. Pair-wise significant differences were determined with *t*-tests or Wilcoxon Mann–Whitney tests. For multiple sample comparison, ANOVA with Tukey’s HSD post hoc analyses were performed in MetaboAnalyst 5.0, and samples were normalized relative to the QC pooled samples. Metabolites were considered differentially enriched if the *p*-value adjusted for false discovery rate (FDR) was less than 0.05. Chemical similarity enrichment analysis was performed using ChemRICH [23], imputing log_10_-transformed concentration data, fold-change values, and *p*-values from *t*-tests. Principal component analysis (PCA) was performed using MetaboAnalyst 5.0.

### 2.9. Antimicrobial Assays

2,4-dihydroxybenzophenone (BP-1, Merck, 818652) and cuminyl alcohol (Merck, 196037) were assessed for their antimicrobial activity. Cell growth (OD_595_) was measured using a microplate-based antimicrobial assay as described previously [16] with modifications. Briefly, BP-1 was solubilized in methanol, and cuminyl alcohol was solubilized in DMSO (both solvents were used at a final concentration below 2%). Metabolites were tested at 25, 12.5, 6.25, 3.125, and 1.562 µg/mL. Each well on the microplate (Greiner Bio-one, item# 655101) contained 98 µL of cells or spores (OD_595_ of 0.01 in Mueller Hinton broth for *S. aureus* and *E. coli*; OD_595_ of 0.001 in YPD for *C. albicans*; 50,000 spores/mL for *F. graminearum* in half-strength PDB) and 2 µL of the tested metabolite. To test whether combining these metabolites had a more potent antimicrobial effect, the assay was also performed using a mix of BP-1 and cuminyl alcohol in equal parts, followed by addition of 2 µL of this mix to each well. Three biological replicates with three technical replicates were prepared for each metabolite or mix and tested against *S. aureus*, *E. coli*, *C. albicans*, and *F. graminearum* (*n* = 2). The contents of the plates were shaken automatically by the microplate reader for 5 s before each read, and the plates were covered with a BreathEasy film (Merck) to prevent desiccation. Bacterial and *C. albicans* plates were incubated for 24 h at 37 °C and 30 °C, respectively. *F. graminearum* plates were incubated for 72 h at 25 °C. After incubation, growth was measured by optical density at 595 nm using a SpectraMax M2 plate reader (Molecular Devices). OD_595_ readings were performed on the day of preparation (t = 0 h) and these values were subtracted from the OD_595_ reading performed at the end of the incubation period. Wells were scanned using a 5-point pattern. Growth was expressed as percentage relative to a control that was treated with DSMO (for cuminyl alcohol) or methanol (BP-1). These controls were considered to have 100% growth.

## 3. Results

### 3.1. Characterization of Extracellular Vesicles from Fusarium graminearum (F. graminearum)

Although a well-described medium for the induction of trichothecenes in vitro for *F. graminearum* has been reported [24], the carbohydrates in this medium interfere with the recovery of EVs from *Fusarium* [10]. Therefore, the medium presented here contained putrescine dihydrochloride and L-arginine to induce the production of trichothecenes and YNB to increase the recovery of EVs from liquid medium; hence, it was named “YNB+putrescine”.

There were no significant differences observed (*p*-value = 0.3) in mycelial weight between cultures grown in YNB+putrescine (6602.5 mg, *n* = 4) and YNB+ without putrescine (5393.8 mg, *n* = 5).

**Figure 1:**
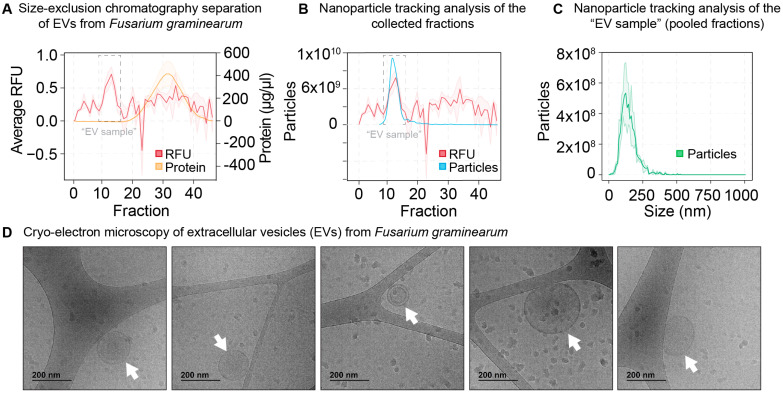
Characterization of extracellular vesicles (EVs) from *Fusarium graminearum* grown on YNB+putrescine liquid medium. EVs were isolated by size-exclusion chromatography. (**A**) Fractions 8–15 typically had the highest relative fluorescence units (RFUs) and were pooled and named “EV sample” (*n* = 8, red line). Soluble protein was determined by micro-BCA and typically eluted in fractions 17–45 (*n* = 2, yellow line). (**B**) Nanoparticle tracking analysis (NTA) revealed a correlation between RFU (red) and particle number (*n* = 1, blue line), as described previously [10]. (**C**) NTA revealed an EV mode diameter of around 120 nm, with an average concentration of 2.38 × 10^10^ particles/L of culture (*n* = 2, green line). (**D**) Cryo-electron microscopy revealed particles (shown with white arrows) with sizes around 200 nm that had a similar morphology to vesicles from other fungi.

EVs were isolated by size-exclusion chromatography. The separation of extracellular vesicles (EVs) from the culture supernatant was monitored by tracking the relative fluorescence units (RFUs) of the hydrophobic dye FM5-95 in each eluted fraction (Figure 1A, red line). Fractions 8–15 typically had the highest RFU, hence these fractions were pooled and named “EV sample”. Nanoparticle tracking analysis (NTA) revealed that the fractions with the highest RFU had the highest number of particles (Figure 1B, blue line) and that the EV sample contained particles with diameters of 30–500 nm and a mode of 120 nm (Figure 1C, green line). The EV sample had an adjusted concentration of 2.38 × 10^10^ particles/L of culture (*n* = 2). Soluble protein was eluted in fractions 17–45 (Figure 1A, yellow line). Cryo-electron microscopy revealed spherical structures that were similar to EVs from other organisms [25,26,27].

### 3.2. Quantification of Zearalenone in Extracellular Vesicle (EV) Samples from Fusarium graminearum by ELISA

**Figure 2:**
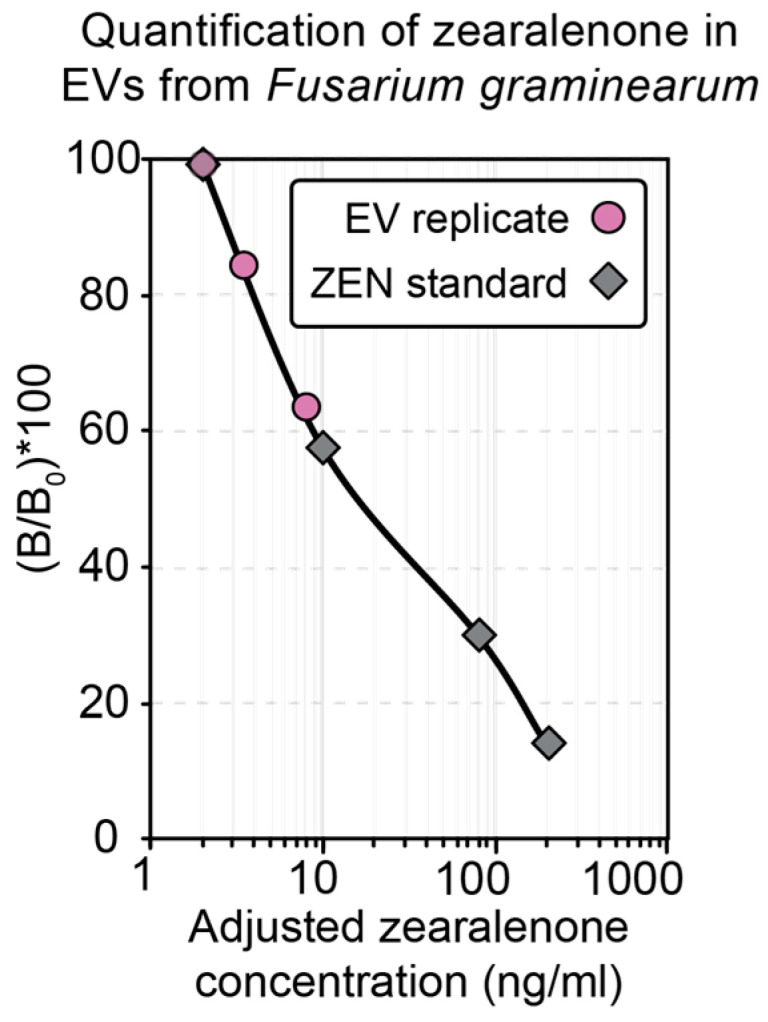
Quantitation of zearalenone (ZEN) in extracellular vesicles (EVs) from *Fusarium graminearum* (*F. graminearum*). ELISA revealed a ZEN concentration of 4.5 ± 1.8 ng/mL (*n* = 3, ± SEM) in EV samples from *F. graminearum*. EV-depleted samples had ZEN levels that were below the detection levels of the ELISA kit employed (Supplementary Table S1).

EV samples returned an adjusted average ZEN concentration of 4.5 ± 1.8 ng/mL (*n* = 3, ± SEM) (Figure 2). The EV-depleted sample, the secretome (SEC), the whole-cell lysate (WCL), the 100 kDa eluate, and PBS produced readings that were below the level of detection of the ELISA kit (Supplementary Table S1). These results revealed that it was appropriate to pursue the characterization of the EV metabolome by LC-ESI-MS/MS.

### 3.3. Extracellular Vesicle (EV) Samples from Fusarium graminearum Contain 2,4-Dihydroxybenzophenone (BP-1) and Cuminyl Alcohol 

**Figure 3:**
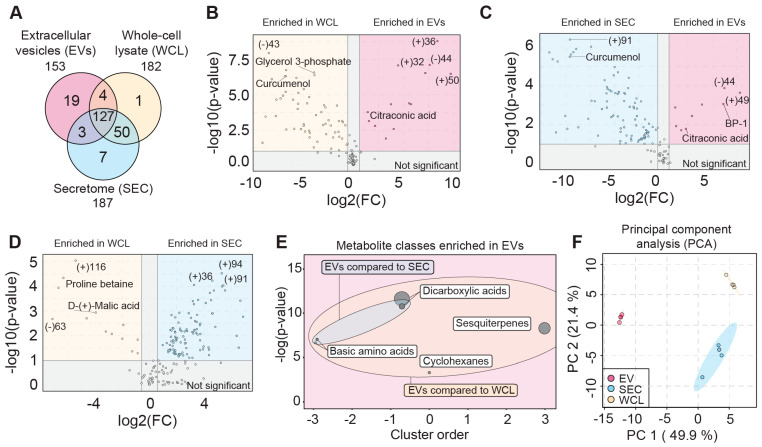
Extracellular vesicles (EVs) from *Fusarium graminearum* (*F. graminearum*) contain enriched metabolites compared with the secretome (SEC) and whole-cell lysate (WCL). LC-ESI-MS/MS metabolomics revealed 153, 187, and 182 metabolites in EV samples, SEC, and the WCL from *F. graminearum*, respectively (**A**). Samples shared 129 metabolites. Volcano plots identified 13 and 9 metabolites that were significantly more abundant in the EVs compared with the WCL (**B**) and the SEC (**C**), respectively, while 102 were more abundant in the SEC compared with the WCL (**D**). (+) and (−) indicate whether the unknown metabolite was detected in positive or negative ion polarity mode. (**E**) The classes of metabolites that were overrepresented in EVs compared to SEC and WCL are highlighted in blue and yellow, respectively. Principal component analysis (PCA) showed good separation and clustering based on sample types and number of biological replicates, respectively (**F**).

The untargeted metabolomic analysis detected 211 metabolites using LC-ESI-MS/MS in the positive and negative ion polarity modes (abbreviated (+) and (−), respectively), with 39 known (18.4%) and 172 unknown (81.5%) metabolites. EV samples, the secretome (SEC), and the whole-cell lysate (WCL) had 153, 187, and 182 metabolites, respectively, and 128 were common to the three samples (Figure 3A).

Of the 153 metabolites detected in EVs, 30 were mass-spectrally identified (19.6%), 123 were unknown (80.3%), and 19 were found exclusively in EV samples (Table 1). The most concentrated metabolite in EVs was the unknown (+) 36 (40,443.30 ng/mL). Further examination by proton nuclear magnetic resonance (^1^HNMR) revealed that this compound was glycerol (Supplementary Figure S1). The second most concentrated metabolite in EVs was 2,4-dihydroxybenzophenone (BP-1) with a relative concentration of 14,249.77 ng/mL. BP-1 was also present in the SEC at a concentration of 18.53 ng/mL and was not detected in the WCL. The most abundant EV-exclusive metabolite was hexaethylene glycol with a relative concentration of 1123.70 ng/mL. Other notable EV metabolites were 8-acetylharpagide (110.67 ng/mL) and cuminyl alcohol (6.73 ng/mL). The metabolite classes that were overrepresented in EVs were sesquiterpenes, dicarboxylic acids, cyclohexanes, and basic amino acids (Figure 3E). The identification of BP-1 and cuminyl alcohol was confirmed by performing targeted LC-ESI-MS/MS using commercial standards (Supplementary Figure S2).

Volcano plots were generated in MetaboAnalysit 5.0 revealing 13 and 9 metabolites that were significantly more abundant in the EVs compared with the WCL (Figure 3B) and the SEC (Figure 3C), respectively, while 102 were more abundant in the SEC compared with the WCL (Figure 3D).

The most abundant known compounds in the SEC were sesquiterpene zedoarondiol, with a relative concentration of 20,887.68 ng/mL, curcumenol (10,709.44 ng/mL), and N,N-dimethylaniline (1250.51 ng/mL). Zedoarondiol was detected in the WCL (2153.69 ng/mL) but not detected in EVs, while curcumenol was less abundant in the WCL (788.58 ng/mL) and least abundant in the EVs (7.30 ng/mL). The most abundant, SEC-exclusive metabolite was “unknown (−) 18” detected at 497.54 ng/mL. D-malic acid was the most abundant metabolite in the WCL with 12,283.68 ng/mL, followed by proline betaine (5796.13 ng/mL) and L-carnitine (2400.67 ng/mL). D-malic acid was also detected in the SEC (793.92 ng/mL) and in the EVs (7.58 ng/mL). L-carnitine was also detected in the SEC (260.41 ng/µL) and was not detected in the EVs. Haplamine was the most abundant WCL-exclusive metabolite at 134.65 ng/mL.

**Figure 4:**
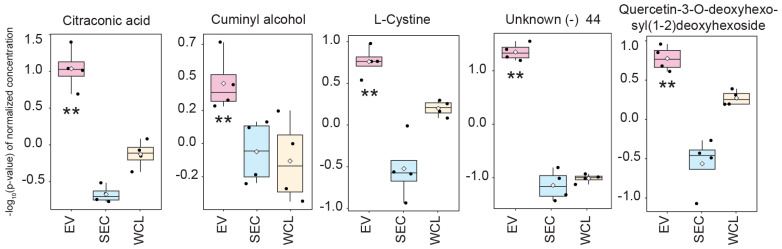
Extracellular vesicles (EVs) from *Fusarium graminearum* are enriched with citraconic acid, cuminyl alcohol, and L-cystine, compared with the secretome (SEC) and whole-cell lysate (WCL). The concentration of the 128 metabolites common to all samples (EVs, SEC, WCL) was analyzed with ANOVA revealing that 42 metabolites are significantly more abundant in EVs in at least one comparison. The known compounds that were enriched in EVs compared with SEC and WCL are presented. Black dots represent the metabolite concentration (ng/mL) of individual replicates (*n* = 4), and the white diamond represents the average concentration; the box and whisker represent the 95% confidence interval around the median concentration relative to the QC pooled samples ± SD. (**) Enriched in EVs compared with SEC and WCL.

Principal component analysis (PCA) revealed clustering of the replicate samples, while the first principal component (PC1) separated the three groups (49.9% variance), indicating good distinction among the sample classes (Figure 2F). The 127 metabolites that were observed in all samples (EVs, SEC, and WCL) were analyzed using ANOVA to identify those that were differentially abundant (Figure 4). Forty-two metabolites (27 known; 15 unknown) were more abundant in EVs in at least one comparison (Supplementary Table S3). Of these, 22 metabolites were more abundant in EVs compared to both the WCL and SEC (Supplementary Table S3). The complete list of metabolites for EVs, SEC, and WCL is available in Supplementary Table S4. The 30 known metabolites detected in EVs were analyzed with ALOGPS 2.1 to predict their hydrophobicity, revealing that 17 out of 31 metabolites (54.8%) had a log10 partition coefficient (logP) above zero, indicating potential hydrophobicity (Table S5).

### 3.4. Metabolites Detected in F. graminearum EVs Reduce the Growth of Bacteria and F. graminearum

Because some of the identified metabolites in *F. graminearum* EVs had previously been reported to have antimicrobial properties, we performed growth inhibition assays against *E. coli*, *S. aureus*, *C. albicans*, and *F. graminearum*, to determine whether components of the EV metabolome could be involved in microbe–microbe interactions or if EVs may be involved in reducing metabolite self-toxicity in *F. graminearum*.

**Figure 5:**
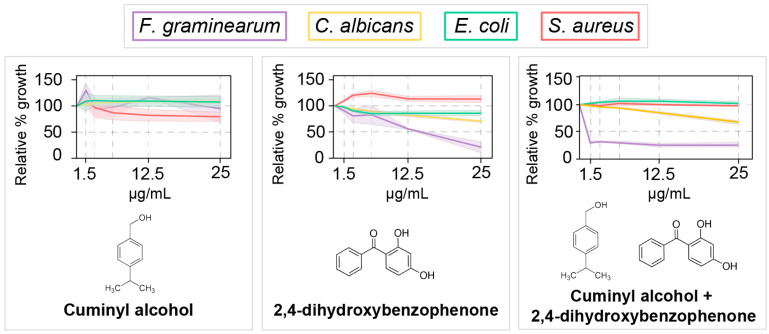
Effect of metabolites detected in extracellular vesicles (EVs) from *Fusarium graminearum* on fungal and bacterial growth. LC-ESI-MS/MS detected metabolites in EVs from *F. graminearum* with previously reported antimicrobial activity. To assess their potential antifungal and antimicrobial roles, the compounds that were commercially available were tested individually as well as mixed against *F. graminearum* (*n* = 2), *Candida albicans*, *Escherichia coli*, and *Staphylococcus aureus* (all *n* = 3) using a microplate-based antimicrobial assay [16]. Cell growth was reported relative to the untreated control and expressed as percentage. 2,4-dihydroxybenzophenone reduced the growth of *F. graminearum* down to about 25% at 25 µg/mL.

The selected metabolites were cuminyl alcohol (found in EVs, SEC, and WCL) and 2,4-dihydroxybenzophenone (BP-1, found in EVs and SEC). Cuminyl alcohol reduced the growth of *S. aureus* to about 80% at 25 µg/mL and did not affect other microbes significantly. At 25 µg/mL, BP-1 reduced the growth of *F. graminearum* to about 25% and *C. albicans* to about 80%; other species were not affected significantly (Figure 5).

## 4. Discussion

Several fungal species, including *Fusarium oxysporum* and *Candida albicans*, have been reported to produce extracellular vesicles (EVs) that enhance their virulence [8,11,28]. The EV proteome of some fungal pathogens has been described, and many of these contain proteins that are likely to support their infection process, such as proteases, cell wall-degrading enzymes, effectors, and polypeptide mycotoxins [8,9,10,29]. However, the composition and putative function of the metabolome from fungal EVs remains understudied.

In this study we investigated whether EVs from *Fusarium graminearum* carry metabolites that could potentially modulate the defenses of the host plant. To characterize the metabolome, we performed untargeted metabolomics using LC-ESI-MS/MS on *F. graminearum* EVs that were recovered from liquid medium (named “YNB+putrescine”) containing known inducers of trichothecene synthesis [24], to determine if these or other virulence factors were present in EV samples.

The YNB+putrescine cultures had an EV concentration about 10 times lower than previous *F. graminearum* cultures grown without putrescine [10]. Interestingly, there were no significant differences in the dry weight of the mycelia between the YNB+putrescine and the YNB+ cultures, suggesting that metabolic or cell environment changes led to the decrease in EV production in this study.

Although putrescine induced trichothecene production in small-scale cultures [24], we did not detect these mycotoxins in our EV preparations using LC-ESI-MS/MS. There are three potential explanations for this. First, there were differences in the medium used by [24] to induce trichothecenes and the medium used in this study to isolate EVs. The first uses a basal medium with sucrose as a carbon source, while the latter uses amino acids as a carbon source because this allows the purification of EVs [10]. Hence, it is possible that *F. graminearum* produces lower amounts of mycotoxins when amino acids are the only carbon source, even with the supplement of putrescine. Secondly, although trichothecenes and zearalenone have been detected in cereals using LC-ESI-MS/MS [30], the inability to detect these toxins in the *F. graminearum* EV samples might be due to the instrumental method set up and extraction protocol used in this study that were not specifically aiming to target these analytes, as mentioned previously. Future research may benefit from developing an LC-MS/MS- or GC-MS/MS-targeted multiple-reaction monitoring (MRM) method, with a validated extraction protocol for these specific metabolites, as well as incubating larger cultures to yield more EV material and incubating for longer times [31].

In contrast to LC-ESI-MS/MS, zearalenone (ZEN) was detected in EVs but not in EV-depleted samples via ELISA. The detection of ZEN by ELISA agrees with previous reports [32]. The presence of ZEN in EVs is supported by the identification of three proteins in *F. graminearum* EVs [10] similar to those found in the zearalenone biosynthesis cluster. These include zearalenone biosynthesis protein 1 (48% identity) [33], a Ca^2+^-transporting ATPase (55% identity) [34,35], and an aldehyde dehydrogenase (29% identity) [33]. Hence, we hypothesize that EVs are involved in the movement of ZEN. A recent report that the mycotoxin fungisporin is transported in EVs from *Penicillium digitatum* [9] supports this hypothesis. Regarding trichothecene detection in EVs, the only deoxynivalenol ELISA kit that could be sourced at the time of this study did not detect DON in *F. graminearum* EV samples This is because the *F. graminearum* strain used in this study (PH-1) produces mostly 15-acetylated DON (15-ADON), and the kit was suitable for detection of 3-ADON. Therefore, future research on *F. graminearum* PH-1 should assess the presence of DON in EVs using a 15-ADON ELISA kit or using mass spectrometry.

The untargeted metabolomic analysis by LC-ESI-MS/MS revealed metabolites in EVs that have been reported by others to have roles in host–pathogen interactions. For instance, 2,4-dihydroxybenzophenone (BP-1), the most abundant known metabolite in EVs, is used as a UV filter in cosmetics, is toxic to crustaceans [36], and damages DNA in mammalian cells [37]. Other fungi, including *Aspergillus* spp., produce benzophenones that have antibacterial activity, inhibit seed germination, and inhibit α-glucosidase [38,39]. These findings agree with our observations that BP-1 reduced the growth of *F. graminearum*. However, since 4,4-dihydroxybenzophenone, a benzophenone similar to BP-1, has been implicated as a potential inhibitor of sterol biosynthesis [40,41], it is possible that BP-1 is transported in EVs as a mechanism to limit self-toxicity. Cuminyl alcohol did not reduce the growth of *C. albicans*, *E. coli*, or *F. graminearum* significantly. Mixing these two EV-enriched metabolites did not result in full inhibition of any of the tested microbes; hence, these may not be antifungal in the conditions of this study, or their real mechanism of action remains to be determined.

While most of the metabolites present in EVs and not in the secretome or the whole-cell lysate are unknown, the function of some of the known EV metabolites has been reported. For instance, the glycoside 8-acetylharpagide inhibits the growth of *Salmonella* and *Klebsiella* [42], and (5E,8Z)-4,7-dihydroxy-2-me-thyl-2,3,4,7-tetrahydrooxecin-10-one (Modiolide A) reduced the motility of *Phytophthora capsici* zoospores [43,44]. Although the function of 7-4′-3′-trimethoxyflavone has not been described in plant–fungal interactions, this metabolite inhibits prostaglandin production and induces wound healing in human cells [45]. Because there is virtually no information on the role of these compounds in the *Fusarium*–plant interaction, the function of these metabolites during fungal infection remains to be determined.

Other metabolites shared in EVs, SEC, or WCL samples included 1,2-cyclohexadione, which acts as a bacterial, fungal, and plant peroxidase inhibitor [46]; dihydroalbocycline, which is an intermediate in the biosynthesis of the macrolide antibiotic albocycline [47]; and mycosporine glutaminol, which is a UV absorbent [48]. However, most of the EV metabolome remains unknown and severely understudied. Further research on this topic can fully elucidate the role of EV metabolites in host–pathogen interactions and may lead to identifying targets to design new antifungals.

The poor water solubility of some of the metabolites found in EVs suggests a role for EVs in the transport of hydrophobic compounds. The lipid component of the EVs, which accounts for about 40% of the EV volume [49], would provide a suitable environment for the transport of these molecules. Indeed, ALOGPS 2.1 predicted that almost 70% of the known EV metabolome may be hydrophobic; hence, it is probable that toxic metabolites, such as BP-1 and ZEN, are concentrated in EVs and potentially delivered to the host. This also highlights the importance of developing mass spectrometry methods and extraction protocols to specifically characterize the hydrophobic metabolites in fungal EVs.

In conclusion, we have performed the first comprehensive metabolomic characterization of EVs produced by a plant pathogen and present a pipeline to study the *F. graminearum* metabolome and perhaps the metabolome of other fungal species, which is an unexploited area of opportunity to find new antifungals. While the virulence-inducing growth medium used in this study produced EVs in lower quantities compared to other media, we were still able to identify hundreds of metabolites. Several metabolites were enriched in EVs, which suggests that EVs play a critical role in metabolite transport and targeting. This study lays the groundwork for a greater understanding of how the metabolites, which are produced and trafficked in the EVs of *F. graminearum*, might interact with their environment.

## Figures and Tables

**Table 1:** Metabolites found in extracellular vesicle (EV) samples from *Fusarium graminearum* and not in the secretome or whole-cell lysate. Untargeted metabolomics revealed 153 metabolites for EV samples. The 19 metabolites that were detected only in EV samples are presented. RT: retention time; Ref. *m/z*: reference mass-to-charge ratio; Av. *m/z*: average mass-to-charge ratio; ng/µL: metabolite concentration in EV samples; RSD: relative standard deviation.

EV-Exclusive Metabolite	RT (min)	Adduct	Ref. *m/z*	Av. *m/z*	Error (ppm)	Formula	Ontology	ng/µL	RSD
Unknown (+) 40	0.606			261.13071				527.51	0.1107
Unknown (+) 44	0.609			155.04849				23.12	0.1944
Unknown (+) 54	0.622			233.04146				17.34	0.0486
Unknown (+) 55	0.629			233.08037				158.07	0.0982
Unknown (+) 56	0.632			289.10645				55.48	0.1317
Unknown (+) 59	0.638			361.16351				18.22	0.1687
Unknown (+) 60	0.641			236.05518				91.78	0.1371
(5E,8Z)-4,7-dihydroxy-2-methyl-2,3,4,7-tetrahydrooxecin-10-one (Modiolide A)	0.643	[M+Na]^+^	237.05237	237.05177	−2.531	C_10_H_14_O_4_	Oxocins	386.11	0.1274
3-(4-chlorophenyl)-2-methyl-5,6,7,8-tetrahydropyrazolo[5,1-b]quinazolin-9(4H)-one	0.646	[M+Na]^+^	314.10532	314.10617	2.706	C_17_H_16_ClN_3_O	Phenylpyrazoles	473.18	0.0865
7,3′,4′-Trimethoxyflavone	0.655	[M+H]^+^	313.10999	313.11029	0.958	C_18_H_16_O_5_	7-O-methylated flavonoids	55.38	0.0849
Unknown (+) 65	0.656			336.08841				19.69	0.1516
Unknown (+) 67	0.656			333.11313				59.93	0.1258
Unknown (+) 68	0.657			191.03159				160.83	0.1826
8-Acetylharpagide	0.658	[M+H]^+^	407.14999	407.14969	−0.736	C_17_H_26_O_11_	Iridoid O-glycosides	110.67	0.0974
Unknown (+) 70	0.659			406.15369				54.08	0.1324
Unknown (+) 72	0.663			415.13553				39.69	0.1305
Unknown (+) 76	0.677			199.02130				13.63	0.1758
Hexaethylene glycol	10.752	[M+H]^+^	283.17511	283.17505	−0.211	C_12_H_26_O_7_	Polyethylene glycols	1123.70	0.1268
Unknown (+) 90	10.754			300.20163				734.73	0.2010

## Data Availability

The metabolomics raw data have been deposited to the MetaboLights [50] database with identified number MTBLS6964.

## Supplementary Materials

The following supporting information can be downloaded at: https://www.mdpi.com/article/10.3390/jof9050507/s1, Figure S1: ^1^HNMR analysis of unknown +(36); Figure S2: MS-MS spectra of BP-1 and cuminyl alcohol; Table S1: Quantitation of zearalenone (ZEN) in extracellular vesicles (EVs) from *Fusarium graminearum* by ELISA; Table S2: EV metabolites; Table S3: ANOVA analysis of common metabolites; Table S4: All metabolites; Table S5: Hydrophobicity prediction for EV metabolites.
